# Medical Items and News of Interest to the Physician

**Published:** 1881-07

**Authors:** 


### For the Use of Physicians.

CULLED FROM THE STANDARD MEDICAL JOURNALS OF THE WORLD.

**Typhoid State**

Dr. J. M. Keating reports a case of pneumonia, in which, in the absence of other stimulant, 3 drops of nitrite of amyl were given in emulsion every three hours, and all other medication stopped The symptoms which called for its administration quickly improved, and this was attributed to the effects of the remedy.—*Medical Times.*

**Asthma.**

Iodide of ethyl has been used successfully by Dr. D. R. Brower (Chic. Med. Jour. and Ex ), after all other remedies had failed. The patient was a boy of fifteen, with an inherited nervous weakness. The dose employed was six drops. The drug relieved the poroxysms and lessened the intervals between them, Dr. Burkhart finds pilocarpin curative in asthmatic cases complicated with chronic bronchitis.—*Independent Practitioner.*

**Styptic Colloid.**

| | |
|---|---|
| Collodion | 100 parts. |
| Carbolic acid | 10 " |
| Tannin | 5 " |
| Benzoic acid (from the gum) | 5 " |

Mix the ingredients in the order above written until perfect solution is effected. This preparation has a brown color, and leaves, on evaporation, a strongly adherent pellicle. It instantly coagulates blood, forming a consistent clot, and a wound rapidly cicatrizes under its protection.

**Pitting of Small Pox.**

Dr. Schwimmer, in the *Gaz. des Hop.*, advises a mask to be formed of very pliable linen cloth, leaving apertures for the eyes, nose and mouth. The inside of this is to be smeared with one of the following liniments: 1. Carbolic acid, 4 to 10; olive oil, 40, and prepared chalk 60 parts. 2. Carbolic acid, 5; olive oil and pure starch, of each 40 parts. 3, Thymol, 2; linseed oil, 40, and chalk, in powder, 60 parts. The mask should be renewed every twelve hours. Compresses, impregnated with one of these mixtures, may also be placed on the hands, and on any parts of the face with which the mask does not come into exact contact.

**Uterine Sedative.**

Dr. Edward C. Mann, of Tarrytown, has had several violent cases of congestive and neuralgic forms of dysmenorrhœa, in one case accompanied with epileptiform convulsions. In each case he "has seen almost magical relief following the use of fluid extract of *Viburnum prunifolium.*" Its use in such cases has, with him, taken the place of everything else. Coincident with the arrest of pain, the nervous excitement, quick pulse, and high temperature have declined.

**Iodoform**

Has been recommended by Dr. Aphel for use in painful local affections. A woman having suffered a bruise of the right mamma, had swelling of the glands in the axilia, and the breast became painful. Twenty days' inunctions of mercury, and belladonna, continued for ten days, gave no relief. The use of an ointment of iodoform gave relief instantly. A man had a bruised ankle, and an ointment of one part of iodoform in 30 parts of lard produced rapid amelioration.

Prof, Marius uses one part of iodoform in thirty parts of glycerole of starch, with enough essence of peppermint to cover the objectionable odor.

**Diphtheria.**

Dr. C. R. S. Curtis, of Quincy, Ill., reports in the *Boston Med. and Surg. Jour.*, the results of the local use of a decoction of leaves of *Juglans nigra* in diphtheria. The remedy was chiefly employed as a gargle or applied with a swab to the throat and fauces. A poultice of the leaves was also resorted to in some instances. Dr. Curtis adopted the practice in consequence of the recommendation by Prof. Nelaton (in his Elements de Pathologie chirurgicale) of the *Juglans nigra* as a remedy in malignant pustule. The use of the gargle was unattended by discomfort, no patient objecting to it; and improvement in each instance was rapid, the oedema subsiding, the ash-colored spots disappearing.

**Gelatine Bougies.**

According to F. Friedrichs, gelatine bougies may be prepared as follows: Melt together on the water-bath 3 parts of white gelatine, 6 parts of glycerine, and 1 part of water. If any remedial agent is ordered to be incorporated, this is, if possible, dissolved in a little water, and added under constant stirring with a glass rod. A

glass tube of proper diameter (about 3 to 5 mm.) is then selected, the interior of which is oiled, and immersed into the melted mass. By gentle suction the tube is filled, immediately closed with the finger, and then allowed to cool, which takes but a short time. An oiled plug is then inserted into one end, and by means of a stout wire or rod the bougie pushed out at the other end. Then it is cut into pieces of appropriate length.—*Pharm. Zeit.*

---

**Freckles.**

The following formula is said to be efficacious for the removal of tan and freckles:

℞

Hyd. bichlor., grs. vj.

Acid. mur. dil,, ℨ j.

Aquæ, ℥ jv.

Alcohol, ℥ ij.

Aq. Rosæ, ℥ ij.

Glycerine, ℥ j.

M. Apply at night and wash off with soap in the morning.—*Canada Lancet.*

---

**Cystitis.**

In a very complete article on cystitis in the Declimnaire Encyclopedique, Dr. Chauvel indicates the following preparations, which have been tried with good results:

℞
Turpentine, 15 grammes.
Camphor, 1 gramme.
Extract of hemlock, 15 centigrammes.

Take a particle as large as a cherry in unleavened bread, morning and evening. Also an old prescription of uva ursi and pareira brava.

---

**Diabetes.**

Dr. Paul Guttmann (Bull. Gen. de Therap., 1880, p. 431: from Berliner Klin. Wochens.), in a case of diabetes, gave for five days no medication. Quantity of sugar, 231.65 grammes. For thirty-one days administration of ammoniacal salts, 223-11 grammes of sugar daily. Then cessation of treatment for thirty-one days. Amount of sugar, 173.19 grammes per diem; thus showing that, so far from diminishing the amount of sugar in the urine, the salts of ammonia actually increase it.—*Phila. Med. Times.*

---

**Antiseptic Catgut.**

Lister prepares catgut for ligatures as follows: Dissolve one part of chromic acid in 4,000 parts of distilled water; add 200 parts of pure carbolic acid; add amount of catgut equal in weight to the carbolic acid; let it remain in the solution 48 hours; remove and dry; then place, until wanted for use, in one-to-five carbolized oil. The gut should be wound about a glass rod or test tube, and the ends fastened, so as to prevent untwisting while soaking and drying.

---

**Albuminous Obstruction of the Bladder.**

Hollmann reports in *Nederl. Wickbl.*, the case of an old man, aged eighty, suffering from retention of urine, in whom the introduction of a catheter failed to procure the desired result. It was found that the bladder contained coagulated albuminoid masses, mixed with blood. A few hours after the injection of about sixteen grains of pepsine dissolved in water, a large amount of a dark, viscid, fetid fluid readily escaped by the catheter.

---

**Toothache.**

Dr. Sporer, of St. Petersburg, uses chloral hydrate in the following manner:

Take three or four small lumps of chloral, wrap them in a little wadding, place this tampon in the hole in the tooth, and let it remain until dissolved. The most severe toothache will disappear in a few minutes under this treatment. —*Druggists Circular.*

---

**Carbonated Laxative Water.**

Take of:

Phosphate of soda.......12 drachms.
Bicarbonate of soda......72 grains.
Water..................20 ounces.
Citric acid..............72 grains.

Dissolve the phosphate and bicarbonate of soda in the water; filter into a stout bottle; add the citric acid; cork the bottle, and tie with cord.

---

**Burns from Sulphuric Acid.**

M. Alanore, a pharmacien of Clermond Ferrand, relates in the *Union Pharmaceutique* the details of an accident by which two students in chemistry received a quantity of sulphuric acid on their faces and clothing. The professor brought them to E. Alanore's pharmacy, and he as quickly as possible covered their faces with a soft paste of calcined magnesia and water, about two millimetres thick. He continued to apply more as this fell off. In a quarter of an hour both were free from pain, but the treatment was continued in one case for five hours, in the other for twenty-four hours. When they were able to bear the air without pain it was found that no mark of the burn remained except a slight redness, which entirely disappeared in the course of a week or two.

**Purgative for Consumptives.**

Dr. Ferraud recommends the following prescription in the constipation of phthisis:

℞

Magnesiæ calcinat., ʒ ss ad ʒ j:

Mannæ, ℥ j ad ℥ ij.

M. To be taken in a cup of tea.

When this is mixed with honey it makes an agreeable electuary:

℞

Mannæ. ʒ j.

Magnesiæ calcinat., ʒ ss.

Mellis, ʒ j.

M. This may be taken in tablespoonful doses in the morning.—*Phila, Med. Times.*

---

**Gastric Flatulence, Acidity and Pyrosis.**

Glycerine does not prevent the digestive action of pepsin and hydrochloric acid; hence, while it prevents the formation of wind and acidity, probably by checking fermentation, it in no ways hinders digestion. One or two drachms may be taken either before, with, or immediately after food; in water, coffee, tea or lemon and soda water. In tea and coffee, it may replace sugar, a substance which greatly favors flatulence, as, indeed, does tea in many cases. In some instances a cure does not occur till the lapse of ten days or a fortnight. Drs. Sydney Ringer and Morrell.—*Kings Co. Proceedings.*

---

**Facial Erysipelas.**

Dr. Rothe observes (*Betz. Memorabilien*) that, however efficacious the subcutaneous injection of carbolic acid proves in arresting the course of erysipelas, it is not suitable when the face is the part attacked, for not only does it give rise to considerable pain, but induces a swollen and painful condition of the periphery. For some years past he has been in the habit of using the following application: Acid. carbolic., sp. vini., *aa,* one part; ol. terebinth. two parts; tinct. iod. one part; glycerin. five parts; pencilling the inflamed skin and its vicinity with it every two hours. No pain or sense of burning is produced, and the skin is usually next day pale and wrinkled. The further progress of the disease is more effectually arrested than by any other remedy, any new patches being rapidly effaced, so that in three or four days the facial erysipelas is usually at an end. The penciled places should be covered by a very thin layer of wadding. When febrile action is present the ordinary internal measures must also be resorted to. —*Med. Times and Gaz., London.*

**Subacute Gout or Rheumatism.**

Dr. Lenoble, of Esternay, has used the following unguent in a case of subacute gout. He has also found it useful in his own case, when suffering on the eighth day from acute articular rheumatism:

℞, Finely-powdered gamboge ..............

Myrrh ...............

Cinnamon ..........

Salicylate of soda..... } aa 10 grammes.

Oil of turpentine... .. q. s.

To be of a fluid consistency.

Three applications should be made daily by rubbing in the preparation briskly, and afterward covering the affected joints with wadding. The same formula will serve neuralgia, recent or of old standing, after the first days of the acute attack have passed.

---

**Ringworm.**

Dr. Malcolm Morris, bearing in mind that thymol has an antiseptic action eight or ten times greater than that of carbolic acid in preventing the development of bacteria, and that chloroform is more rapidly absorbed than alcohol, probably owing to its being more readily taken up by the sebaceous glands, has tried with success, the use of thymol, or menthol, mixed with chloroform, with oil added to hinder the rapid evaporation of the latter, and to prevent it from acting as an irritant. The proportions used are thymol or menthol, ½ drachm (or less when the skin is tender or irritable); chloroform, 2 drachms; olive oil, 6 drachms. He states that in this way the remedy is more easily absorbed, and actually reaches and destroys the fungus in the hair follicles.

---

**Urticaria Produced by the Use of Tincture Pimpinallæ.**

Dr C. Kaufmann, of Frankfurt am Main, relates a case of which a brief account is given in *Allgemeine Medicinische Central-Zeitung,* February 26, 1881, in which he ordered twenty drops of tinct. pimpinallæ every three hours, in sweetened water, for the relief of a slight angina. The patient, a girl of sixteen, complained the following day that she had been unable to sleep all through the night, on account of an uncomfortable burning and itching of the skin, and also stated that there was an eruption. Upon examination it was found that the arms, hands, chest, back and abdomen were covered with a rash, while the lower extremities were entirely free. There was no general disturbance and the urticaria disappeared by evening, upon the withdrawal of the medicine and dusting the parts with starch powder. Two days later the medicine was again administered, with the same result as before.

**Prolapsus Ani.**

R. Eichler, M.D., in *Western Lancet*, says: A boy five years of age came under my treatment, suffering from prolapsus ani of two years standing. The gut came out to the extent of two and a half inches after each passage. My treatment at first was of the routine kind—cold effusions, cauterizations with nitrate of silver, tincture of iron, etc. The bowel persisted in coming down at every passage. As a last resort, I tried an ergotine suppository.

℞ Ergotine.................. gr. ij
But. cocoa................. q. s.
M. Ft. Suppos............. no. j

One after each passage.

The effect of the remedy has been magical, as after the use of a few of the suppositories, there has been no return of the condition, and the case is cured.

**Tic Douloureux.**

Doctor Fereol of Lariboisiere, has used Ammonio-Sulphate of Copper in four cases of tic douloureux, with results so satisfactory, that he strongly recommends its re-introduction. He prefers the following formula :

B

Cupric-ammonio-sulphat., 1½-2 grs.

Syr., ℥ j.

Aq., ℥ iij.

M. This quantity is to be taken during the 24 hours, preferably after vegetable food. If the pain continue, increase the dose. In one case as much as nine grains were used during the day, giving rise, however, to gastro-intestinal disturbance; even the administration of the usual doses will cause fetor ex ore and a metallic taste, nevertheless continue with 1½ gr. daily for 12 to 14 days.—*Med. Times and Gazette—Norwegian Journal of Medicine.*

**To Keep Leeches.**

Perhaps your readers would like to know a way to keep leeches with the least trouble, and always have them healthy.

Take any wide-mouth bottle that will admit the hand, and fill it about two-thirds full of what is known as "Excelsior" (such as is sometimes used in upholstering and making cheap matresses), wash the "Excelsior" with warm water and pour it off; then pour in cold, soft water enough to cover, and put in the leeches, tie a piece of thin cloth over the top, change the water once a month, and occasionally set the bottle and contents in the sun.

I have used this method for a number of years, and I do not remember ever finding a dead leech. It has certainly proved better than any jar, sponge, rusty nails, earth, or anything else I ever tried, and has the recommendation of being cheap and easily attended to.

JAMES S. TALBOT.

DORCHESTER, April 14th, 1881.

**Sodium Phosphate in Habitual Colic.**

Dr. R. N. Taylor writes in the *Medical Herald:*

"It has fallen to my lot to have been beset with a number of patients who would persist in having colic at the most inopportune moments imaginable; first one, and then the other, then still another one, these colicky friends of mine were constantly interfering with my professional engagements, and what was more important still, with my hours of rest. Without entering into a discussion of the pathology of these cases, I beg leave to present in brief the history of one of the worst I have ever seen, together with the treatment, which resulted in completely warding off the attacks in this case, as in all others in which it has been carried out:

"Mrs H., aged seventy-two years, for fifteen years has been the subject of attacks of cramp colic, recurring at first every two or three months, but for six years past coming on every two weeks, sometimes every week; has taken nearly everything in the materia medica, both at the hands of regulars and quacks, without any benefit, the attacks continually recurring, and that without any regard to errors of diet, coming on at nearly regular intervals, no matter how careful or how particular she might be in regard to her diet.

"During the paroxysms of colic the pain is most atrocious, accompanied by cramping pain in the extremities, nausea, vomiting, etc., to such an extent as to demand my immediate presence, armed with the hypodermic syringe.

"This patient was put upon the use of phosphate of soda, grs. xxx., ter in die, before meals and—the fees stopped! She had no more attacks of colic. If she omits the use of soda for a length of time, say six or eight weeks, she will have a few premonitory twinges, the precursors of a more severe attack; but immediately obtaining a supply of the drug, she is safe so long as she continues to use it, and for some time afterward.

"This is not an isolated case, but one of several that could be adduced in favor of the use of phosphate of soda in the colic habit; but it is deemed useless to multiply instances upon so trivial a matter. Suffice it to say that I have never failed to see the administration of phosphate of soda followed by a complete cessation of the attacks of colic, and my experience in the use of this drug has now become quite extensive.

"In these cases I generally begin with thirty grains three times a day, and if that amount produces much irritation of the bowel, indicated by frequent small discharges, attended perhaps by some tenesmus, I diminish the dose to twenty or fifteen grains. It is to be administered before meals, from a half to one hour, in a glass of water, and when thus dissolved it is not at all unpleasant to the taste."

---

**Tape Worm.**

Dr. Martiali, chief of sanitary affairs in Senegal, reports in the *Annales de Medecine navale*, that the cocoanut has been well known to the people of the Antilles since the time of the aborigines. It was used by him successfully in more than nine cases. The method of using it is as follows: The cocoanut is divided into small pieces, grated and eaten slowly by the patient. After three hours a dose of castor oil is taken, and the worm will pass away after five or six hours. The action of the remedy is similar to that of pumpkin seeds. ANOTHER.—If a tænia is placed in a solution of pepsine in a glass, it is entirely dissolved—digested in a few hours. Guided by this fact, M. Beuchut says in *Lyon Medical* that he treated a child with pepsine, who had, under treatment with pumpkin seed, passed a piece of tapeworm about eighteen inches in length and very broad. The dose given was about 45 grains per diem, for five days. The child experienced no harm and showed no special symptoms. Then he was given 40 centigrammes (about seven grains) of sulphate of pelletierine with castor oil, which was followed by the expulsion of no section of tænia. The author adds that this question should be investigated anew.

Since then, the same author has again employed, not animal pepsine, but that derived from vegetables—papaine—which is much more active, and claims to have relieved several children by its use. One child passed fragments of tapeworm ten inches in length, softened, yellowish, withered and partially digested.

These cases, added to those observed in the colonies, where the juice of the carica papaya is employed successfully against worms, prove that this new substance may well be used, in future, against verminous affections.

---

**Acute Rheumatism.**

Prof. M. Charteris, M. D., of Glasgow University, writes to the *British Medical Journal:*

Experimentally, I tried, four years ago, in two cases of equal gravity, salicylate of soda and salicin; and I found that the former produced decidedly alarming and toxic results, while the latter excited its harmless and beneficial influence. I was so much impressed at the time with what I witnessed, that I resolved never to give the artificial compound again in the disease, but to trust to the natural product, and in doing so I have been led to formulate the following conclusions:

1. In uncomplicated cardiac cases, salicin in doses of twenty-five grains, dissolved in warm water or milk, will lower the temperature in forty-eight hours.

2. When this has been accomplished, diminish the frequency of the repetition of the dose mentioned to every six hours; and finally, after two days, stop it altogether, as its further continuance is useless and depressing, and retards convalescence.

3. If the temperature be not lowered in the time mentioned, in all probability the heart is affected; and if such should be the case, the medicine should be countermanded, for it will then in no way diminish the fever. The cardiac complication may not be detected by auscultation at the time; but it will invariably be found that a cardiac murmur exists at the natural termination of the disease. For this cardiac complication I apply a large blister over the region of the heart, and do not allow it to heal quickly.

3. Previously to employing salicin, I test the urine for albumen; and if this be detected, and if there be a suspicion of old standing kidney disease as revealed by the history of the patient, it is probable that the drug may not be properly eliminated from the system. That it is so can be readily seen by testing the urine at intervals by tincture of perchloride of iron, when a purple color will be observed, owing to the action of the iron transformed in the system into salicylic acid. Should this reaction not be seen, it is well to discontinue the use of salicin.

5. Finally, I may state that I have never in any case found salicin to cause thirst or delirium. These phenomena may be attributed to impurities in the artificial compound—probably to carbolic acid. Salicin seems to me to have some special action in rheumatism, for it will be found not to lower the temperature in other affections where this is high. Thus, in a case of advanced phthisis it is inert in large doses, while quinine, in similar circumstances, exerts its usual antipyretic virtues when given at a different time to the same patient.

In a recent visit I made to some of the chief continental hospitals, I found that the pure salicin was now alone employed, and, I may add, implicitly believed in, for acute rheumatism.

**Perforation of Membrana Tympani from Ascaris Lumbricoides.**

On March 3d I was called to see a woman. aged thirty-five, three or four months advanced in pregnancy. She was in a low nervous state, and had been suffering since Christmas from nausea and vomiting. About two weeks before she had vomited several round worms, and about the same time suffered severely from dyspepsia, and intense pain in the chest and abdomen. Shortly before my arrival she had vomited two, and three more discharged from the nostrils, her nose bleeding first for three hours. I prescribed four grains of santonine powder, to be taken at bedtime; after taking which four worms were passed per rectum, for the first time. The santonine was followed next morning by fifteen grains of compound scammony powder, when a great many more were passed per rectum. Three or four hours after taking the second powder, and having previously suffered all night from intense earache, a neighbor discovered a worm protruding from each ear and both ears bleeding; the same day three others came away from the ears, two from the left and one from the right. The following morning, March 5th, her husband drew another from the ear, and again on March 8th; this last was four inches long with the diameter of a small goose quill. A large number were also discharged each day by the bowel, making in all seventy-four.

*Remarks.*—The history of this case appears at first incredible; but there can be no doubt the membranes were perforated by the passage of the ascarides. In addition to being vomited, some must have crawled up the æsophagus into the fauces, thence some found their way into the nasal passages, and others into the Eustachian tubes, perforating the tympanic membranes and being discharged by the external auditory meatus.—*Lewis W. Reynolds, M. R. C. S. Eng., Complete in London Lancet.*

---

**Backache; Its Cause.**

Dr. George Johnson, in (*Brit. Med. Jour.*) says that in the great majority of cases the pain of backache has its seat in the muscles, and is a simple result of strain or over-fatigue of the lumbar and erectores spinæ muscles and tendons. A remarkable feature of the pain resulting from excessive muscular exercise is that, while it may continue more or less during rest in bed, it is usually much increased by the first movements after rest, but gradually diminishes after moderate exercise. In muscular lumbago, standing is more fatiguing for the back and legs than walking, and leaning forward puts a greater strain on the muscles of the back than standing erect. Pain is often more severe on one side than on the other, owing to the common practice of throwing the weight more on one leg than on the other. A common cause of painful over-strain in the dorsal muscles is an excessive weight in the abdomen, whether from advanced pregnancy, dropsy, or excessive development of fat. Dr. Johnson, in speaking of these causes, incidentally gives the dietary advisable in obesity. He also calls attention to dyspeptic myalgia resulting from malnutrition of the muscles. "Growing pains," Dr. Johnson thinks, are due to excessive muscular exercise, and are to be cured by rest. Sudden pain is sometimes caused by cramp or rupture of some fibers of the muscle during contraction. Indigestible food sometimes cause cramps in muscles in certain individuals instead of cramps in the stomach. Cold, as is well known, is a frequent cause of pains in other muscles besides those of the back. For lumbago Dr. Johnson recommends hot air or Turkish baths, followed by vigorous shampooing; also an embrocation composed of equal parts of linimentum belladonnæ and linimentum opii.

Other causes of backache are aneurism of the aorta, the symptoms of which Dr. Johnson gives at some length, with illustrative cases ; cancerous glands in the abdomen; disease of the kidneys; gastric ulcer ; uterine diseases ; diseases of the bones of the spine and of the spinal cord, and, finally, the backache of commencing fevers.

**Pilocarpine**

Has been recommended by Dr. George Guttmann, of Cronstadt, as a remedy in diphtheria and other inflammatory affections of the tonsils, pharynx, and larynx. In six cases of diphtheria, its use was followed by recovery in two to four days.

For children he employed the following formula:

Muriate of pilocarpine, gr. $\frac{1}{6}$ to $\frac{1}{3}$.
Pepsin, gr. x. to xxij.
Hydrochloric acid, gtt. ij,
Distilled water, ℥ viij.

A teaspoonful to be taken hourly.

For adults:

Muriate of pilocarpine, gr: $\frac{1}{2}$ to $\frac{3}{4}$.
Pepsin, gr. xxx.
Hydrochloric acid, gtt. iij.
Distilled water, ℥ viij.

A teaspoonful hourly.

He is accustomed to give a small amount of good wine after each dose.—*Berliner Klin. Woch.*

**Vaccine Virus.**

In a brief review of a late discussion on vaccination by the Medical Society of Kings county, among other things, Dr. Burge says:

"The records of the New York Dispensary and Kine-pox Institution are also appealed to in the report, as showing an average of one case of erysipelas in every five hundred vaccinations with humanized virus, where no cases had ever come to Dr. Martin's knowledge out of thousands of persons known to him to be vaccinated with true animal virus.

Now, it does not seem to me at all surprising that one case of erysipelas should have happened in five hundred vaccinations in the N. Y. Dispensary—indeed, one-fifth of one per centum seems a small proportion, when we consider the character of the children who apply to such an institution. It may also be safely assumed that the atmosphere and general conditions favorable to the development of erysipelas are present both in a dispensary where fifty thousand patients a year are treated, and in the homes from which these patients come. I have vaccinated with humanized virus at least two thousand children, and have never in my own practice seen a case of erysipelas follow the operation. My own conclusion, therefore, is that if the source from whence the virus is taken, and the time and manner of taking it, and the method of its introduction, are all right, and erysipelas follow, we may be absolutely certain that the evil arises from the condition of the child, or his surroundings, and not from the virus or the operation, except, indeed, as an exciting cause.

The first rule laid down by your Committee on Hygiene is 'Vaccinate with only pure virus.'

Now, what is pure virus? We are all, of course, familiar with the fact that the original source was a vesicle upon the udder of a cow, which had a disease taken in the *natural* way. In the early days of my practice, when I heard occasionally of a resort to the bovine virus, I was simple enough to suppose that somebody hunted around among the fat and lean kine until he found one with the characteristic eruption of vaccinia, and that the market was supplied from this stock. This, however, was not the case; but *calves* were inoculated with what? Sometimes with virus from other calves, and sometimes with the very virus which some suspected had lost its virtue, and the ridiculous assumption reached that the resulting virus would not only be better than the seed, but as good as the original stock. This, however, does not represent the case in all its absurdity. We know from analogy that the perfection of vaccine must depend upon its being taken the natural way.

The first method of lessening the virulence of small-pox was by insufflation. How long this practice was in use in India before the method of inoculation with variolous matter came into vogue we do not know, but this second method was equally successful, and both methods vitiated the disease, as they were intended to do. Now if we institute an interminable series of inoculations among the calves, how long will it be before the vaccine disease is so modified, or mollified, that we cannot expect it to hold its place as the greatest of all prophylactics?

What virus have we now in use? Probably six or seven kinds: 1. We have, I hope, though I don't know where to obtain it, virus direct from the natural vaccinia. 2. We have humanized virus originally from the natural bovine vesicle. 3. A virus resulting from humanized virus introduced again to the system of the cow, and brought back to man. 4. Probably a virus resulting from inoculation of the cow with small-pox matter. 5. It is not impossible that we have a virus from other vesicular diseases in the cow, resembling vaccinia. 6. We have the calf to calf virus, originally from the true natural kine-pox, and now descended through a long line of induced cases of vaccinia."—*Complete in Kings Co. Proceedings, Brooklyn.*

